# Emergence: Complexity Pedagogy in Action

**DOI:** 10.1155/2015/235075

**Published:** 2015-03-09

**Authors:** Christine Jonas-Simpson, Gail Mitchell, Nadine Cross

**Affiliations:** ^1^School of Nursing, Faculty of Health, York University, Toronto, ON, Canada M3J 1P3; ^2^York-UHN Academy, University Health Network, 200 Elizabeth Street, Toronto, ON, Canada M5G 2C4

## Abstract

Many educators are looking for new ways to engage students and each other in order to enrich curriculum and the teaching-learning process. We describe an example of how we enacted teaching-learning approaches through the insights of complexity thinking, an approach that supports the emergence of new possibilities for teaching-learning in the classroom and online. Our story begins with an occasion to meet with 10 nursing colleagues in a three-hour workshop using four activities that engaged learning about complexity thinking and pedagogy. Guiding concepts for the collaborative workshop were nonlinearity, distributed decision-making, divergent thinking, self-organization, emergence, and creative exploration. The workshop approach considered critical questions to spark our collective inquiry. We asked, “What is emergent learning?” and “How do we, as educators and learners, engage a community so that new learning surfaces?” We integrated the arts, creative play, and perturbations within a complexity approach.

## 1. Introduction

Sir Robinson [1] calls for a revolution in education founded on three principles: diversity, curiosity, and creativity. He suggests that we in education need to create a broad curriculum that is open to diversity where students are free to be creative and awaken their imaginations and where teachers facilitate learning rather than seeking compliance. Similarly, Thomas and Seely Brown [2] challenge educators to explore how one's passion can deepen learning. We could not agree more. A learning revolution is* critical for higher education*, and we propose that complexity thinking, with a focus on emergence, may liberate education to support meaningful changes in teaching-learning. Concepts affiliated with complexity thinking inform the “how to” bring about a learning revolution where diversity, curiosity, passion, and creativity are at the fore. For example, complexity curriculum concepts such as Doll's 4 Rs (richness, recursion, relations, and rigor) [3, 4] and the notion of* perturbation* [5] can open students to the edge of their abilities rather than facilitating or making easy their experience; it is through perturbation that students' thinking grows [6, p. 33]. Diversity, emergence, curiosity, creativity, perturbation, richness, recursion, relations, rigor, and passion all align with a complexity thinking approach to teaching and learning [3, 7–10].

There is a paucity of literature on complexity science in nursing and yet Davidson et al. state that “the concepts within the science of complexity will shape the future of nursing inquiry, practice and education” [11, p. 17]. Some literature that describe concepts from complexity in education was found in nursing [11, pp. 372–374], medicine [12], dentistry [13], and interprofessional training [14]. Given this gap in explicating how complexity can help change education, we offer one example of how we enacted a collaborative inquiry of complexity thinking with faculty colleagues, which surfaced in the collective new possibilities for teaching and learning in the classroom. The integration of the arts, creative play, and perturbations within a complexity approach is shown. Let us turn to our example.

## 2. The Workshop: Creating Spaces of Possibility

This story begins with an opportunity to engage with 10 nursing colleagues in a three-hour workshop using four activities that engaged learning about complexity thinking. As a group of three—call us* perturbers* rather than facilitators—we met to discuss possibilities for the workshop. Our own teaching, research, and leadership have changed with complexity thinking [15–18]. We share, with other nursing authors [19–22], the deep dissatisfaction with content-driven curricula where teachers dispense information in a linear format with predetermined learning outcomes [3, 23]. The concern with this content-driven, dispensing model is that students learn to look for what teachers want in order to give it back without necessarily learning how to learn, think, critique, and engage with ideas and each other. The idea of creating spaces of possibility for learning together surfaced and resurfaced at our planning meetings and continued to emerge during our online communications about how we believed the workshop might unfold. Oriented by complexity thinking we are comfortable with nonlinear processes, ambiguity of learning outcomes, and distributed control and decision-making [8–10]. We are also inspired by the belief that

a successful collectivity is not just more intelligent than the smartest of its members, but that it presents occasions for all its members to be smarter—that is, to be capable of actions, interpretations, and conclusions that none would achieve on her or his own [7, p. 136].

We decided to approach the workshop with our colleagues as a collaborative inquiry. We also considered critical questions [24] that we believed would spark our inquiry for the three-hour workshop. We asked “What is emergent learning?” and “How do we, as educators, engage a community so that new learning surfaces?” The following situates our thinking about emergent learning.

## 3. What is Emergent Learning?

In contrast to andragogy (self-directed learning) and heutagogy (self-determined learning), complexity pedagogy (relational learning) supports emergent learning in a collective [10, 23, 25]. We purposely set up the activities for our workshop to facilitate emergent learning, that is, new learning that emerges from the collective conversations and activities. This learning is not conceptualized as coconstructed with reflective thoughts of each participant as constructivist pedagogies purport [26]; rather, the learning emerges out of a diverse community in a way that is unpredictable and prereflective. In 2004, Davis et al. wrote of emergence as “instances of webbed, nested, multilayered narratives that become more intricate dense and full of possibility” [25, p. 4]. It is through conversation that learners engage and transform with differences so that emergence reflects a deep and more personal learning [8, 23]. It is the engagement with diverse perspectives/knowledge that unsettles, complicates, perturbs, and calls for further conversation [5, 10]. Each emergent learning moment is unpredictable and unique and cannot be predicted or predetermined beforehand.

So, how do educators set up situations of possibility such that new learning emerges? That is one question we asked and, through our own conversations and tossing ideas, we came up with some strategies for possible ways to engage with colleagues. We decided to work with three strategies that we believed would be most likely to create spaces of possibilities and emergent learning: integrate metaphor and use a non-linear design and collective activities. We want to speak briefly about each of these strategies beginning with metaphor.

## 4. Use of Metaphor to Open Collective to the Whole

With the essential elements for emergent learning [7, 8] in mind, we invited participants to read one or more of the three readings we chose based on complexity thinking and education: Doll [3], Menin [12], and Mitchell et al. [16]. We also asked each participant to come to the workshop with a metaphor that reflected her experience of teaching-learning. Metaphors defy boundaries, reflect the whole, and are consistent with high level thinking complexity pedagogy [27]. Menin further describes the power of metaphor for learners:

Effective teaching makes use of metaphors for the description of complex events because metaphors both constrain and liberate learners. They attract diverse learners to a collectivity while simultaneously tolerating the ambiguity of creative freedom necessary for connections with the metaphor [12, p. 163].

We began our workshop discussion by asking colleagues to share their teaching-learning metaphors during their introductions to the group. As noted by Carter and Pitcher [27], metaphors are particularly helpful for engaging faculty in complex dialogues. Participants' metaphors for teaching-learning included roller coasters, being in a forest but unable to see the trees, the act of swimming, a colorful vortex designed snail, and space travel. Many participants referred to their own metaphor, as well as to others, during the introductions and throughout the 3-hour workshop, which reflected the infinite recursiveness that metaphors can bring to a learning community. The second strategy we focused on for creating spaces for emergent learning was a nonlinear design for the workshop.

## 5. Workshop Design

We designed our workshop with a desire for openness and creativity. The workshop activities were set up in nonlinear stations or spaces; that is, we asked colleagues to work as a collective to decide what station they would visit first and* how* they wanted to engage with each of the station activities. We anticipated that station activities would enable colleagues to think in different ways, that is, more collectively, conceptually, and/or artistically and more divergently from usual patterns of teaching-learning. It was also our intent to support flexibility while providing sufficient opportunity to hold the group's creativity in meaningful ways. After each activity we returned to our circle to engage in a collective conversation of specific ideas and perturbations that were intentionally crafted to “bump” into one another [6, 7, 28]. We believe the conversations were playful, valuing of difference, and a place where we were, for those conversational moments, relinquishing the certainty of our usual teaching practices [4]. We engaged as a collective knowledge-generating system, believing that the emergence of new learnings would arise. New understandings continued, through the recursive movement between an activity and coming together in conversations and with perturbations. After the conversation following the fourth activity, we revisited the concept of* emergence* and asked participants to record new critical questions, aha moments, understanding, puzzlements, and perturbations. A final discussion ensued, guided by a line of creative inquiry [29].

Prior to describing the final discussion, we turn to the four activities that included completion of a teaching-learning narrative; assembling fractal art puzzles; an exploration of liberating-constraints; and selection of complexity concepts. Recall that our colleagues were asked to consider the four activities and chose how and in what order to engage them. We stayed in somewhat of an observant stance with our colleagues as they considered the activities and began a process of engagement.

## 6. Activities Designed for Emergent Learning

### 6.1. A Teaching-Learning Narrative

This activity involved completing a teaching-learning narrative while considering complexity concepts learned from the prereadings. We asked participants to find a partner and finish the following narrative integrating some notion or understanding that they garnered from readings on complexity pedagogy while also considering what they already knew about complexity pedagogy.

It's the first day of Fall term and I have to enter a class with 150 third year nursing students and I am full of both dread and excitement. The course is on community health and I am most concerned about helping students to see a different view of community and how they might start a health-promoting activity. I have prepared one main activity to kick things off with this group and I hope it sets us on a path of conversation and learning. I decided I would….

After the pair completed the narrative, they were asked to return to our conversation circle and share what they wrote and what complexity idea(s) they were trying to capture. The following questions were prepared to start the discussion.

 What complexity ideas do you see connecting together in your stories?
 How do some of these ideas connect with your teaching-learning experiences?
 What is intriguing to you about complexity thinking and pedagogy?

With the narrative activity our colleagues engaged with different complexity ideas and how they might get enacted. Researchers have documented favorable outcomes when faculty create and participate in critical and reflective dialogues about teaching [30, 31]. Participants heard various strategies to teaching-learning from one another, as we all explored the inventive side of pedagogy when education is not prescriptive but open and engaging.

### 6.2. Fractal Art Puzzles

Another activity involved fractal art puzzles. Fractals offer visual representation of self-organization, recursiveness, and beauty. We gathered images of fractals and cut images into pieces and asked the group to see how many picture puzzles they could solve as a group in the time allotted. The group of 10 participants decided to work on the fractals as a whole. How they chose to reassemble and reimagine the art was up to them; however, they were constrained to the cut-up pieces of fractals provided to them. Some group members moved to rearranging the pieces while others commented and advised over shoulders. A few decided that it would be interesting to combine two of the fractals to create a new entity.

Three of the four puzzles were easily solved as participants recognized the self-similar patterns. But the fourth puzzle was more complex with so many self-similar patterns that colleagues could not make sense of the whole. Some people had the hands on action while others made suggestions from outside the circle. The fractal was not successfully reconstructed but it created a great deal of discussion, laughter, and learning as a question arose, “Does every puzzle have an answer?” It also surfaced the realization that sometimes patterns are not recognized, and the larger whole can be illusive and hidden. And yet, it is there, waiting to be seen.

With the intent of exploring the learning of the collective after the fractal activity, the following questions were asked when we reconvened in our conversation circle.

- How did you work together as a group to solve the puzzles?
- What was it like to work in relationship and to respond to this inquiry of fractal art?

Participants described their own preferences of being with the puzzle-solving activity. They observed how some faculty jumped right in to start rearranging puzzle pieces while others preferred to stand back and watch colleagues to see if different insights came with a distant view. The fractal puzzles provided some creative time with visual symbols of complexity thinking and to invite the group to think about how to work to solve these puzzles in community. Our next activity addressed the important notion of liberating-constraints and how there need to be both freedom and restriction in complexity pedagogy.

### 6.3. Liberating-Constraints Exploration

For this activity we provided a definition of liberating-constraints and then asked the group members to think of specific teaching-learning activities they would create to set up a liberating-constraint in one of their courses. Informed by Davis et al. [8] and Newell [32], we defined liberating-constraints as the boundaries that shaped our purpose for coming together. Liberating-constraints provide a shared understanding for what direction to pursue without prescribing how the group works together. For example, students may be given an assignment to create engaging health education for any audience using teaching-learning concepts and the arts. The students are free to choose the topic for health education, the audience, and the art-form medium; however, they are required to use teaching concepts and the arts in their assignments.

Participants were asked to return to the circle to share a specific teaching-learning activity they would create to set up a liberating-constraint in one of their courses. Our intent with this activity was to give colleagues a chance to hear different understandings and ways of creating enabling-constraints in a teaching-learning environment. Our activities for the workshop were examples of liberating-constraints and ones our colleagues could experience firsthand.

### 6.4. Concepts of Complexity Thinking

The faculty workshop was about complexity thinking in teaching-learning that sets the constraint, the boundary that establishes content [28]. We decided to offer a tabletop filled with complexity concepts and to ask colleagues to choose two concepts that they were most interested in. We included the following concepts:

 Reflection/Thinkering/Transformative/Emergence/Interruption/Interplay/Possibilities/Insights/Web-like/Conversation/Boundaries/Nonlinearity/Self-Similarity/Interconnections/Recursion/Perturbation/Relational/Patterns/Both-And.

The freedom to choose personal concepts of interest is the liberating aspect within the constraint of the topic of complexity. Participants were asked to return to the circle and describe the following.

- How did you choose your complexity concepts/ideas?
- How do your concepts/ideas relate with the readings provided or your teaching-learning?
- What concept resonates with you most from the readings?

With this activity participants engaged with the content of complexity (the constraint) within the relevant context or personal interest of each participant. If this is a way of creating space of possibility in a workshop in complexity, might a similar process work in the classroom? All in all, the workshop was a study in self-organization and we turn to that idea now in relation to complexity and pedagogy.

## 7. Learning and Self-Organization

Doll states that complexity theory, itself, is “the study of self-organizing systems” [3, p. 26]. Self-organization is a complex phenomenon that emerges when participants come together and share a common space for learning [7–9]. Turning to our example, the four activities were placed at the end of the table and by the windows in a large seminar room. When we asked the group to go to the other end of the room and explore the activities and choose the order in which* the collective* would engage with each, an initial uncertainty and tentativeness ensued. For Doll, “this intermediate situation between order and chaos is where self-organization occurs” [3, p. 22]. Indeed the group gathered around the four activities and with some discussion they decided to engage as one group with activity two where they were required to assemble fractal art. The group movements and organization were not dictated by a central organizer, but rather they emerged from the collective. Unexpected possibilities emerged as the group moved through the activities in a way that was clearly collective-directed within the constraints of the workshop. There were several examples of identifying and sharing new ideas for teaching-learning arising from the circle conversations throughout the workshop, with many aha moments. A great deal of laughter and enthusiastic energy filled the room and a feeling of camaraderie emerged, troubling the feeling that teaching had to be a sole and lonely endeavor. As facilitators/perturbers, we are familiar with complexity thinking and the premise of self-organization; however, it is living in the presence of this process where the power of collective learning is reawakened for us.

## 8. Occasions for Emergence

Supporting diverse views in which learners engage and transform with differences [7–10] and creating boundaries with freedoms, in other words, liberating-constraints [3, 7–9], were two of the ways we intentionally crafted our workshop while enacting complexity thinking as teachers-learners. Each of these processes supported our expectation for emergence and can be designed to some extent, only.

Participants shared ideas with one another creating a space for* collaborative learning* and we tried to invite the diverging views that enrich collective learning. Participants had similar and different experiences in teaching-learning and these differences added depth to the collective inquiry. One participant shared how she fosters diversity in her classes by asking, “Who can offer a different viewpoint?” It was evident that there were some diverse ideas and yet many were consistent with one another. Another way to invite expression of diversity is to ask persons to give a specific example or situation to elaborate. Teachers can introduce diversity by contributing diverging theoretical or philosophical frames that add layers of possible meaning and interpretation. Supporting divergent views occurred amid the emergent design of each activity which both liberated and constrained the participants' learning. Creative play and the arts were used as a way of enabling the participants' individual, paired, and collective imaginations. We turn now to the closing conversations, which provided an opportunity for sharing and explication of emergent learning.

## 9. Explicating Emergence through Closing Conversations

Closing conversations opened us to new collective possibilities and were guided by a form of inquiry based on the work of Springgay et al. [29] to provoke conversation. The following questions were asked so our colleagues and we could reflect upon the experience of the workshop as a whole.

 What ideas stand together or connect for you from the ideas we discussed today?
 What are some ideas about complexity and teaching-learning that you had not connected before today?
 What did you not notice before this workshop experience?
 What ideas linger for you about complexity and pedagogy?
 How did the ideas ripple or reverberate among us? Of what else do the ideas remind you?
 As you listen to each other, what new thoughts stay with you?
 What is lingering in your thinking as an unanswered question or perplexity?
 What feelings do you have about complexity pedagogy?

Participants described enjoyment and excitement with the collective process of making decisions, trying to solve puzzles, and sharing personal experiences. A number of faculty members commented on how academic life can be isolating and competitive. In contrast, the collective freedom to engage and play with ideas about how teaching can be enhanced was both enlightening and satisfying. The metaphors shared at the beginning of the workshop resurfaced in the final discussion in comments such as “I now see there are others swimming with me” and “complexity thinking helps you see the forest and the trees.” The exploration of individual concepts was meaningful as the faculty, as a group, expressed their own understandings of concepts they were interested in taking to their classrooms. For instance, one faculty imagined a group activity where individual students would organize themselves to present different perspectives of familiar health concerns. Faculty indicated a new appreciation for the place of divergence, not only to be more inclusive and critical but also to evoke a deeper exploration into values and assumptions.

We believe that new insights into possibilities for teaching-learning emerged in this workshop guided by a complexity lens and a shared commitment to diversity, creativity, and curiosity. Most significant for all of us was the emergence of shifting patterns of isolation to patterns of relating and collaboration, revealing that the traditional academic culture of competitive individualism can be transformed to a pattern of collective learning and creativity. This paper presents ways to enact complexity ideas with the hope that more systematic explorations in teaching-learning will evolve in coming years that better evaluate the efficacy of complexity for engagement and learning.
